# Forward genetics identifies HN1L/JPT2 as a novel carboplatin resistance gene in ovarian cancer

**DOI:** 10.1016/j.gendis.2025.101720

**Published:** 2025-06-13

**Authors:** Han Wei, Aishat Motolani, Kenneth P. Nephew, Guanglong Jiang, Faranak Alipourgivi, Steven Sun, Xiumei Huang, Mateusz Opyrchal, George Sandusky, Yunlong Liu, Tao Lu

**Affiliations:** a Department of Pharmacology and Toxicology, Indiana University School of Medicine, Indianapolis, IN 46202, USA; b Melvin and Bren Simon Comprehensive Cancer Center, Indiana University School of Medicine, Indianapolis, IN 46202, USA; c Medical Sciences Program and Department of Anatomy, Cell Biology & Physiology, Biology Building 302, Indiana University School of Medicine, Bloomington, IN 47405, USA; d Department of Medical & Molecular Genetics, Indiana University School of Medicine, Indianapolis, IN 46202, USA; e Department of Radiation Oncology, Indiana University School of Medicine, Indianapolis, IN 46202, USA; f Department of Medicine, Division of Hematology/Oncology, Indiana University School of Medicine, Indianapolis, IN 46202, USA; g Department of Pathology and Laboratory Medicine, Indiana University School of Medicine, Indianapolis, IN 46202, USA; h Department of Biochemistry and Molecular Biology, Indiana University School of Medicine, Indianapolis, IN 46202, USA

Ovarian cancer (OC) is the deadliest gynecologic malignancy, with platinum-based chemotherapy, such as carboplatin, remaining the standard first-line treatment. However, resistance to carboplatin poses a major therapeutic challenge, and its mechanisms are not fully understood.^1^ This study employed a novel validation-based insertional mutagenesis (VBIM) technique^2^ to identify genes driving carboplatin resistance in human epithelial OC cells. Our screen identified hematological and neurological expressed 1-like (HN1L/JPT2) as a novel contributor to resistance. HN1L overexpression increased resistance to carboplatin, whereas shRNA-mediated knockdown sensitized OC cells to treatment. Mechanistically, HN1L conferred resistance by activating nuclear factor κB (NF-κB) signaling. HN1L depletion also reduced anchorage-independent growth *in vitro* and tumorigenicity in an OC xenograft model. Immunohistochemical analysis revealed elevated HN1L expression across multiple stages of OC in both cell lines and patient tissues. Collectively, our findings identify HN1L as a previously unrecognized carboplatin resistance gene and suggest that targeting HN1L may offer a promising combination strategy with carboplatin for overcoming platinum resistance in OC.

The VBIM technology^2^ employed in this study utilizes high-titer viral vectors to achieve efficient insertion into mammalian genomes, resulting in robust gene overexpression. A2780 cells infected with VBIM and treated with carboplatin (1 μM) for 2 weeks yielded resistant colonies, indicating mutation-driven survival. Surviving cells were purified, and VBIM-specific cDNA sequencing identified HN1L as the sole gene. Cre recombinase reversed the phenotype, confirming VBIM insertion as the cause. PCR verified HN1L overexpression, validating its role in carboplatin resistance (Fig. 1A, B).

**Figure 1:**
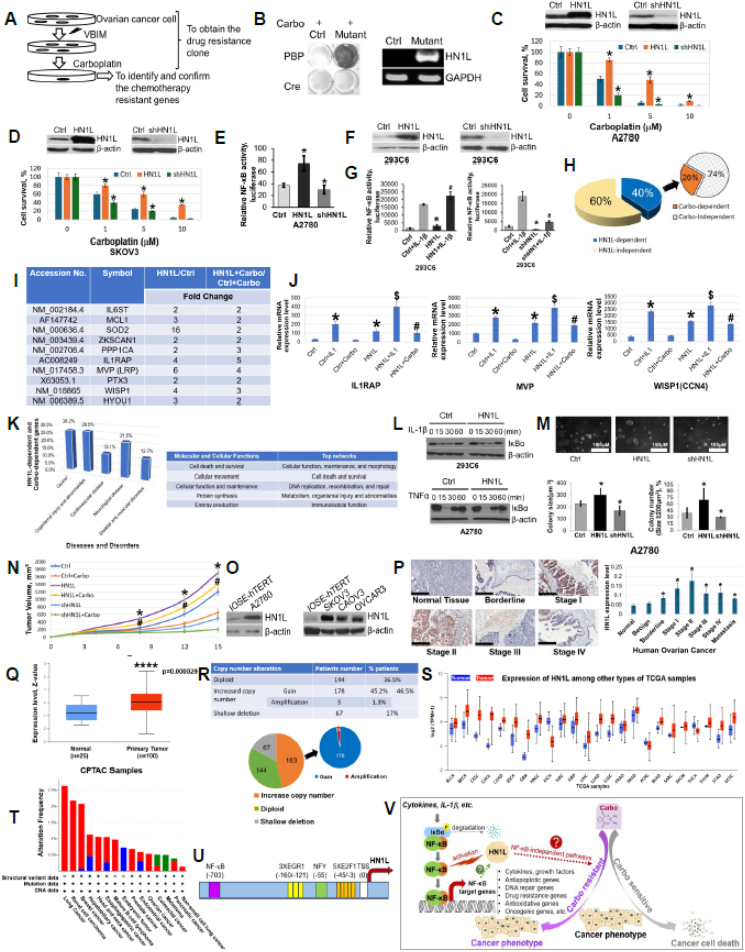
VBIM identifies HN1L as a novel mediator of Carbo resistance via NF-κB activation in OC. **(A**–**D)** Identification of HN1L as a Carbo resistant gene in OC. (A) The schematic showing how VBIM technique is used to identify a chemoresistant gene from OC. Briefly, OC cells are infected with VBIM viruses, which may randomly drive the overexpression of chemoresistance genes, such as Carbo resistance genes. Infected cells are then treated with chemotherapy drugs, such as Carbo, thus, OC cells without Carbo resistance will be killed, leaving only OC cells that harbor the overexpression of Carbo resistance to survive. These cells will be further expanded, and the Carbo resistance genes that are overexpressed driven by VBIM technique will then be identified and further confirmed. (B) Discovery of HN1L as a Carbo resistant gene in OC cells. Left panel: Cells that harbor Carbo resistance survive under the treatment of Carbo, while OCs cells without Carbo resistance die. Cells are stained by methylene blue. Right panel: Inverse PCR with VBIM primers, the band in mutant identified HN1L as the Carbo resistance gene from the Carbo resistance OC cells. (C, D) Functional confirmation of the Carbo resistant gene HN1L in OC cells. Top panel: Western blots, showing HN1L overexpression (HN1L) and knockdown (shHN1L) in OC A2780 cells (C) or SKOV3 cells (D). Bottom panel: Cell viability was determined by clonogenic assay. Overexpression of HN1L resulted in increased cell survival as compared with control A2780 cells (C) or SKOV3 cells (D) under the treatment of a range of concentrations of Carbo, while knockdown of HN1L showed the opposite effect. Results shown are representative of three independent experiments. ∗*P* < 0.05, HN1L overexpressing cells or shHN1L cells vs. Ctrl; bars, standard deviation. **(E**–**G)** HN1L positively regulates NF-κB transcriptional activity. (E) NF-κB luciferase assay, showing that HN1L affects NF-κB activity in A2780 cells. Compared with control, HN1L overexpression increased NF-κB activity, while shHN1L decreased the NF-κB activity. (F) Western blots, showing HN1L expression level in 293C6 cells after transfection. HN1L expression is either increased under HN1L overexpressing condition or decreased under shHN1L condition. (G) NF-κB luciferase assay**,** showing overexpression of HN1L significantly increased the activation NF-κB induced by IL-1β (left panel), while greatly decreased upon shHN1L knockdown (right panel). Results shown are representative of three independent experiments. ∗*P* < 0.05, HN1L vs. Ctrl or shHN1L vs. Ctrl. ^#^*P* < 0.05, Ctrl + IL-1β vs. HN1L + IL-1β or vs. shHN1L + IL-1β. **(H**–**L)** Regulation of NF-κB-dependent gene expression by HN1L. (H) Illumina array data from 293C6 HN1L overexpression cells (labeled as HN1L in the analysis) treated with IL-1β or Carbo. 293C6 cells were used as the control (Ctrl). The pie chart showed that among all NF-κB-dependent genes (818 genes) in response to IL-1β treatment, 40 % (330 genes) of them were HN1L-dependent (solid blue color), among which, 26 % of them (86 genes) were Carbo-dependent (solid orange color). (I) A short list of typical NF-κB-dependent genes that are both HN1L-dependent (HN1L/Ctrl ≥2) and Carbo-dependent (HN1L + Carbo/Ctrl + Carbo ≥2). (J) Confirmation of Illumina array data by quantitative PCR analysis, showing overexpression of HN1L further enhanced IL-1β-induced target gene expression (IL1RAP, MVP, WISP1). Furthermore, Carbo treatment enhanced HN1L-induced target gene expression. Results shown are representative of three independent experiments. ∗*P* < 0.05, Ctrl + IL-1β or HN1L vs. Ctrl; ^$^*P* < 0.05, HN1L + IL-1β vs. HN1L; ^#^*P* < 0.05, HN1L + Carbo vs. Ctrl + Carbo. (K) Ingenuity pathway analysis (IPA), showing HN1L-associated top diseases and disorders (left panel), and molecular and cellular functions as well as top networks (right panel). (L) HN1L regulates NF-κB activity independently from IκBα. Top panel: 293C6 HN1L overexpressing stable cell lines were treated with IL-1β for different time points; cell lysates were probed with IκBα antibody. No significant IκBα degradation difference was observed between these two groups. Bottom panel: Similar IκBα degradation result was observed in A2780 and A2780 HN1L overexpressing cells treated with TNFα, suggesting HN1L activates NF-κB activity independently from IκBα degradation. **(M, N)** HN1L overexpression promotes tumor growth and increases Carbo resistance. (M) Colony formation in A2780 cells, showing HN1L overexpression promoted colony formation in soft agar. The scale bar is 100 μm. Bottom left panel: Average size of colonies in Ctrl, HN1L and shHN1L cells. Bottom right panel: Colony number (%) of the colonies with size bigger than 200 μm. Statistical analysis of average colony size and colony number (%). ∗*P* < 0.05 vs. Ctrl group. (N) HNL1 overexpression promotes tumor growth and increases Carbo-resistant A2780 xenograft tumor growth (*n* = 6/group, 4-to-5-week-old female NSG mice). ∗*P* < 0.05, HN1L overexpression (labeled as HN1L) vs. Ctrl; ^#^*P* < 0.05, HN1L + Carbo vs. Ctrl + Carbo. **(O–R)** HN1L expression is increased in OC tissues compared with normal tissues. (O) Western blots, showing that compared with normal control IOSE-hTERT, HN1L was universally overexpressed in OC cells. (P) Examples of HN1L protein expression levels observed in OC tumor tissue array (TMA) by immunohistochemistry (left panel), showing HN1L was broadly overexpressed at OC tissues compared with normal control. Quantification of staining is shown in the right panel. This TMA array contains 100 samples. ∗*P* < 0.05, cancer samples vs. normal tissue (tumor adjacent normal tissue). Magnification: 20 × . Scale bar: 200 μm. (Q) HN1L (JPT2) is significantly overexpressed in primary OC patients as compared with normal group. Z-values represent standard deviations from the median across samples for the samples. Log2 Spectral count ratio values from The National Cancer Institute's Clinical Proteomic Tumor Analysis Consortium (CPTAC) were first normalized within each sample profile, and then normalized across samples. ∗∗∗∗*P* = 0.000029, primary OC group vs. normal group. Data resource: UALCAN: https://ualcan.path.uab.edu/cgi-bin/ualcan-res.pl. (R) Putative copy-number alterations of HN1L (JPT2) of OC patients from GISTIC (Genomic Identification of Significant Targets in Cancer) (data resource: cBioPortal). Top panel: Table, showing 46.5 % OC patients have increase copy number of HN1L, among these, most of the cases are gain of copy number (178 out of 183 cases, 97.3 %), and a few (5 cases) are of amplification. Bottom panel: Pie graph, showing putative copy-number alteration of 394 OC patients. **(S–U)** HN1L is highly expressed among most types of cancer samples. (S) UALCAN data indicates that besides OC, HN1L also has significantly higher expression in most types of cancers. Normal (blue), Tumor (red) (Data resource: UALCAN: https://ualcan.path.uab.edu/cgi-bin/ualcan-res.pl). (T) Genetic alterations of HN1L across cancer types. Quite some cancer types show increased amplification of HN1L genes (Data resource: cBioPortal, combined study from 10 PanCancer studies with 76,639 samples). (U) Predicted potential transcriptional factors located in HN1L 1 Kb promoter region: E2F1, NFY, EGR1, and NF-κB that may drive HN1L expression. Graph shows how the potential transcriptional factors are located. **(V)** Hypothetical model, illustrating that HN1L overexpression confers Carbo resistance via NF-κB activation in OC. In classical NF-κB pathway, in response to cytokine like IL-1β, IκBα will be phosphorylated and degraded. Thus, NF-κB is liberated and translocated into nucleus to bind to the κB consensus sequence and activate its target genes transcription. In OC cells with significant higher HN1L expression, HN1L would further augment the activation of NF-κB, thus, inducing NF-κB target genes, like cytokines, growth factors, antiapoptotic genes, DNA repair genes, drug resistance genes, antioxidative genes, oncogenic genes, etc. Together, these genes would collectively function to further promote OC tumor phenotype, and lead to Carbo resistance in OC. Additionally, it is possible that HN1L is further induced by activated NF-κB, thus, forming a feedback loop, to further enhance the Carbo resistance phenotype. Finally, it is also possible that, in addition to NF-κB pathway, HN1L may also regulate NF-κB-independent pathway(s) to contribute to Carbo resistance. These interesting aspects warrant further exploration in the future. VBIM, validation-based insertional mutagenesis; HN1L, hematological and neurological expressed 1-like; NF-κB, nuclear factor κB; OC, ovarian cancer; IL-1β, interleukin-1beta; IL1RAP, interleukin 1 receptor accessory protein; MVP, major vault protein; WISP1, WNT1 inducible signaling pathway protein 1; IκBα, nuclear factor κB inhibitor alpha; BLCA, bladder urothelial carcinoma; BRCA, breast invasive carcinoma; CESC, cervical squamous cell carcinoma; CHOL, cholangiocarcinoma; COAD, colon adenocarcinoma; ESCA, esophageal carcinoma; GBM, glioblastoma multiforme; HNSC, head and neck squamous cell carcinoma; KICH, kidney chromophobe; KIRC, kidney renal clear cell carcinoma; KIRP, kidney renal papillary cell carcinoma; LIHC, liver hepatocellular carcinoma; LUAD, lung adenocarcinoma; LUSC, lung squamous cell carcinoma; PAAD, pancreatic adenocarcinoma; PRAD, prostate adenocarcinoma; PCPG, pheochromocytoma and paraganglioma; READ, rectum adenocarcinoma; SARC, sarcoma; SKCM, skin cutaneous melanoma; THCA, thyroid carcinoma; THYM, thymoma; STAD, stomach adenocarcinoma; UCEC, uterine corpus endometrial carcinoma; E2F1, E2F transcription factor 1; NFY, nuclear transcription factor Y; EGR1, early growth response 1; Carbo, carboplatin.

To assess HN1L's role in carboplatin resistance in OC, A2780 and SKOV3 cells were engineered to overexpress or knock down HN1L. Clonogenic assays showed that HN1L-overexpressing cells were significantly more resistant to carboplatin, while shHN1L cells were more sensitive. IC50 values in A2780 cells were 1 μM (control), 4.8 μM (HN1L), and 0.5 μM (shHN1L); in SKOV3 cells, 2 μM, 6.8 μM, and 0.8 μM, respectively. These findings suggest HN1L overexpression enhances carboplatin resistance in OC cells (Fig. 1C, D).

NF-κB signaling has long been associated with chemoresistance, including resistance to platinum agents.^1^^,^^3^ We thus investigated whether HN1L modulated NF-κB activity. Luciferase reporter assays in A2780 and 293C6 cells revealed that HN1L overexpression significantly enhanced NF-κB activity, both basally and in response to interleukin-1beta (IL-1β), while HN1L knockdown suppressed it (Fig. 1E–G). These findings indicate that HN1L acts as a potent activator of NF-κB signaling.

Microarray analysis of 293C6 cells identified 818 NF-κB–responsive genes upon IL-1β stimulation, 40% of which were further up-regulated by HN1L. Approximately 26% of these co-regulated genes have previously been implicated in platinum resistance (Fig. 1H). Quantitative PCR confirmed the up-regulation of key NF-κB targets, including interleukin 1 receptor accessory protein (IL1RAP), major vault protein (MVP), and WNT1 inducible signaling pathway protein 1 (WISP1) in cells overexpressing HN1L and treated with IL-1β (Fig. 1I, J). These genes are associated with inflammation, drug efflux, and tumor progression.^4^ Network analysis (Fig. 1K; Fig. S1) revealed that these genes participate in DNA replication, repair, and metabolic pathways, with NF-κB at the core of the regulatory hub.

To explore the mechanism of NF-κB activation, we analyzed IκBα (nuclear factor κB inhibitor alpha) degradation following HN1L overexpression. Western blotting showed rapid IκBα degradation within 15 min of IL-1β treatment and recovery by 60 min, indicating that HN1L amplifies NF-κB activity, potentially through pathways downstream of IκBα (Fig. 1L).

*In vitro*, HN1L overexpression promoted anchorage-independent growth in soft agar, whereas knockdown suppressed it (Fig. 1M). *In vivo*, HN1L overexpression enhanced tumor growth and conferred carboplatin resistance in xenograft models (Fig. 1N). Immunohistochemical analysis of OC tissue microarrays (*n* = 100) and cell lines revealed significantly elevated HN1L expression across all clinical stages (Fig. 1O, P), further supporting its role in OC progression and resistance.

Proteomic data from The National Cancer Institute's Clinical Proteomic Tumor Analysis Consortium (CPTAC) confirmed significant HN1L overexpression in primary OC compared with normal ovarian tissue (*p* = 0.000029) (Fig. 1Q). Additionally, copy number analysis from Genomic Identification of Significant Targets in Cancer (GISTIC) and cBioPortal revealed amplification of the HN1L gene in a large subset of OC cases (Fig. 1R). UALCAN data further showed increased HN1L expression across multiple cancers, including colorectal and lung cancers (Fig. 1S). Copy number alterations were also observed in various tumors, many involving gene amplification (Fig. 1T).

To investigate transcriptional regulation of HN1L, we analyzed its promoter and found predicted binding motifs for E2F transcription factor 1 (E2F1), nuclear transcription factor Y (NFY), and early growth response 1 (EGR1). Notably, an NF-κB p50 binding site was identified at −703 bp (Fig. 1U), raising the possibility of a transcriptional feedback loop wherein NF-κB could drive HN1L expression, further amplifying its own activity.

Based on our data, we propose a model (Fig. 1V) in which HN1L promotes carboplatin resistance through activation of NF-κB signaling. Under normal conditions, IL-1β stimulation leads to IκBα degradation, permitting NF-κB nuclear translocation and target gene transcription. When HN1L is overexpressed, NF-κB activation is further amplified, leading to increased expression of target genes, which promote cellular survival, reduce apoptosis, and enhance drug efflux or DNA repair, thus facilitating chemoresistance.

Importantly, our findings do not exclude additional mechanisms of action. Whether HN1L influences other pathways such as phosphoinositide 3-kinase (PI3K)/protein kinase B (AKT), or WNT remains to be investigated. For example, hematological and neurological expressed 1 (HN1), a homolog of HN1L, regulates glycogen synthase kinase 3 beta (GSK3β) phosphorylation via PI3K/AKT and has been implicated in drug resistance. HN1 is also among a panel of four genes that can distinguish OC from normal ovarian tissue and may serve as a diagnostic biomarker.

Our VBIM technique enables discovery of genetic changes influencing cellular phenotypes. Previously, we identified NF-κB regulators F-box and leucine-rich repeat 11 (FBXL11)^2^ and armadillo repeat-containing protein 4 (ARMC4). In this study, VBIM uncovered HN1L as a carboplatin resistance gene, offering new insights into drug resistance mechanisms in OC. Previous studies have implicated HN1L in other cancers as well. In breast cancer, it promotes migration and correlates with poor clinical outcomes.^5^ Its silencing resensitized chemoresistant triple-negative breast cancer to docetaxel. In non-small cell lung cancer, HN1L drives cell cycle progression via the mitogen-activated protein kinase (MAPK) pathway. These findings suggest a conserved oncogenic function for HN1L across multiple malignancies.

The NF-κB target genes up-regulated by HN1L in this study are implicated in multiple cancer hallmarks. IL1RAP plays a key role in inflammation and is overexpressed in various solid and hematological cancers. MVP is a vault complex protein associated with non-P-glycoprotein-mediated multidrug resistance.^4^ WISP1, a WNT pathway gene, has emerged as a promising plasma biomarker for early OC detection in independent cohorts.

Interestingly, HN1L expression was highest in early-stage (I–II) OC, and somewhat lower in late-stage (III–IV) disease (Fig. 1P). This suggests that HN1L may be especially important in early resistance development, although reduced expression in later stages could result from tumor microenvironmental influences or other factors. These intriguing observations merit further investigation.

In summary, using the VBIM screening platform, we identified HN1L as a novel gene driving carboplatin resistance in OC. Mechanistically, HN1L activates NF-κB signaling, resulting in the up-regulation of pro-survival and drug resistance genes. HN1L functions downstream of IκBα and promotes tumorigenesis both *in vitro* and *in vivo*. It is overexpressed and frequently amplified in OC and other malignancies. Future studies should investigate potential feedback loops involving NF-κB and explore whether HN1L influences additional oncogenic pathways.

Collectively, our findings establish HN1L as a promising diagnostic biomarker and therapeutic target. Targeting HN1L, alone or in combination with carboplatin, could help overcome platinum resistance in OC and potentially in other HN1L-driven cancers.

## CRediT authorship contribution statement

**Han Wei:** Writing – original draft, Investigation, Formal analysis, Data curation. **Aishat Motolani:** Writing – original draft, Formal analysis. **Kenneth P. Nephew:** Writing – review & editing. **Guanglong Jiang:** Formal analysis, Data curation. **Faranak Alipourgivi:** Writing – review & editing. **Steven Sun:** Writing – review & editing. **Xiumei Huang:** Writing – review & editing. **Mateusz Opyrchal:** Writing – review & editing. **George Sandusky:** Methodology, Investigation, Data curation. **Yunlong Liu:** Writing – review & editing. **Tao Lu:** Writing – review & editing, Writing – original draft, Supervision, Project administration, Funding acquisition, Conceptualization.

## Funding

This work was supported by the US National Institutes of Health (No. 1R01GM120156-01A1, R03CA223906-01, 1R03CA283225-A1 to T.L.), the Kay Yow Cancer Fund (V Foundation for Cancer Research) (USA) (No. 4486242 to T.L.), Showalter Scholar fund from Indiana University School of Medicine (USA) (No. 2286263 to T.L.), and the Indiana University Melvin and Bren Simon Comprehensive Cancer Center (IUSCCC) fund (USA) (No. 2987664 to T.L.).

## Conflict of interests

As a member of Editorial Board of *Genes & Diseases*, Tao Lu has no involvement in the peer-review of this article and has no access to information regarding its peer-review. Other authors have no competing interests to declare.

## References

[1] Eskander R.N., Moore K.N., Monk B.J. (2023). Overcoming the challenges of drug development in platinum-resistant ovarian cancer. Front Oncol.

[2] Lu T., Jackson M.W., Singhi A.D. (2009). Validation-based insertional mutagenesis identifies lysine demethylase FBXL11 as a negative regulator of NFkappaB. Proc Natl Acad Sci U S A.

[3] Momeny M., Yousefi H., Eyvani H. (2018). Blockade of nuclear factor-κB (NF-κB) pathway inhibits growth and induces apoptosis in chemoresistant ovarian carcinoma cells. Int J Biochem Cell Biol.

[4] Szaflarski W., Sujka-Kordowska P., Pula B. (2013). Expression profiles of vault components MVP, TEP1 and vPARP and their correlation to other multidrug resistance proteins in ovarian cancer. Int J Oncol.

[5] Jiao D., Zhang J., Chen P. (2021). HN1L promotes migration and invasion of breast cancer by up-regulating the expression of HMGB1. J Cell Mol Med.

