# Supplementary material for: Forward genetics identifies HN1L/JPT2 as a novel carboplatin resistance gene in ovarian cancer
Source: Genes Dis. 2025 Jun 13;13(1):101720. doi: 10.1016/j.gendis.2025.101720 (PMC12466132; doi:10.1016/j.gendis.2025.101720)
Supplement: Multimedia component 1 [file mmc1.docx]

**MATERIALS AND METHODS**

**Cell lines and materials**

A2780 and SKOV3 were purchased from ATCC. OVCAR-3 and Caov-3 were obtain from Indiana Clinical and Translational Sciences Institute specimen storage facility. Immortalized ovarian surface epithelial (IOSE) was gift from Dr. Daniela Matei from Indiana University School of Medicine. A2780, SKOV3 and OVCAR-3 were cultured in RPMI-1640 medium with fetal bovine serum to a final concentration of 5%. Caov-3 was cultured in DMEM with fetal bovine serum to a final concentration of 5%. IOSE cell cultured in medium consisting of 1:1 MCDB 105 medium and Medium 199 with 10% fetal bovine serum. Lipofectamine and PLUS Reagents were purchased from Invitrogen. TRC Lentiviral Human HN1L shRNA (pool of 5 shRNAs) was purchased from Thermo Fisher Scientific. Ovary disease spectrum (ovarian cancer progression) tissue microarray was purchased from US Biomax, Inc. Anti-HN1L antibody was purchased from Abcam, anti-IκBα was from Santa Cruz anti–β-actin was from Sigma. Carboplatin was purchased from Sigma.

**VBIM technology**

The VBIM technology has been described previously by Lu et al.^17,18^ Briefly, the vector has lentivirus backbone and splice donor sequence, and LoxP sites on both ends. Libraries of cells are generated using VBIM, with integrations of a strong CMV promoter randomly into the genomes of mammalian cells. The promoter stimulates high expression of downstream gene. A2780 cells were infected with VBIM virus to cause the overexpression of drug resistance genes, and then were further under 1μM carboplatin treatment. Using VBIM specific primers, we identified the target gene of interest.

**Clonogenic cell survival assay**

Cells were plated in triplicate per data point into 6-well plates, and were treated with carboplatin (0, 1, 5 and 10μM), then cultured at 37°C for 9 days. Colonies were fixed with 3:1 methanol/acetic acid and stained with crystal violet (Fisher). Colonies were counted using the inverted microscope (AmScope), with a cut-off of 50 viable cells. The surviving rate was calculated and clonogenic survival curves were then plotted. Experiments were repeated at least 3 times.

**Transfection and luciferase assay**

Lipofectamine and PLUS Reagents (Invitrogen) were used for transfection. To establish stable pools, cells were co-transfected with a plasmid encoding a puromycin resistance gene followed by selection in 1 μg/mL of puromycin. For NF-κB luciferase assays, the κB-luciferase construct p5XIP10 κB^17^ and β-galactosidase construct were cotransfected transiently into the cells and luciferase activity was assayed 48 h later. β-galactosidase activity was used to normalize for transfection efficiency. Transfections and luciferase assays were carried out essentially as described by Lu et al.^17^

**Western analysis**

Cells were cultured to 95% confluence, and samples were collected and assayed by the Western blotting as described by Lu et al.^17^

**Immunohistochemical analysis**

Ovarian cancer tissue microarray was obtained from US Biomax Inc. [Rockville, MD]. Hematoxylin-eosin [H&E] was performed using routine methods. The tissue microarray has 100 samples. It was blocked with protein blocking solution [Dako Corp.]. All subsequent staining steps were performed using the Dako FLEX SYSTEM on an automated Immunostainer. Incubations were done at room temperature and Tris buffered saline plus 0.05% Tween 20, pH 7.4 [Dako Corp] was used for all washes and diluents. Anti-HN1L primary antibody was used to detect HN1L localization, followed by Horseradish peroxidase conjugated secondary antibody and the addition of the chromogen. The Aperio whole slide digital imaging system was used for imaging and quantification. The system imaged all slides at 20X.

**Illumina microarrays**

Illumina TotalPrep RNA Amplification Kit [Ambion/Applied Biosystems] was used to reverse-transcribe RNA [250 ng] into cDNA, which then hybridized to Illumina Human Ref-v3v1 Expression BeadChips and scanned in a BeadArray Reader using standard protocols [provided by Illumina]. Illumina BeadStudio software was used for data analysis. This work was with help from University of Chicago Genomics Facility.

**Quantitative PCR analyses**

For quantitative PCR (qPCR) experiments, 293C6 and 293HN1L stable cell lines cultured to 80–90% confluence were treated with IL-1β (10ng/ml, 4h) and with/without carboplatin (40uM, 24h). TRIZOL reagent (Invitrogen) was used to extract RNA, which was further purified by the RNeasy Kit (Qiagen). SuperScript III First-Strand Synthesis System (Invitrogen) was used to synthesize cDNA from total RNA of 293C6 cells. FastStart Universal SYBR Green Master ROX (Roche) was further used for the qPCR reactions. All primers were designed by Primer Express 3.0 software.

**Anchorage-independent (Soft agar) assay**

Type VII agarose [Sigma] was autoclaved and used. Cell culture dishes were coated with 1.2% type VII agarose (mixed with DMEM) as the bottom layer. Cells were resuspended in 0.6% of type VII agarose and plated on top of the bottom layer. Cells were then cultured for 2-3 weeks before being checked under a microscope, measured and quantified using ImageJ software.

***In vivo* xenograft experiments and treatment schedule**

A2780, A2780-HN1L and A2780-shHN1L cells were counted, resuspended in 200 mL 1:1 RPMI-1640/Matrigel (BD Biosciences), and 7 X10^5^ cells were injected subcutaneously (s.c.) into the right flanks of 4- to 5-week-old female NSG mice (NOD.Cg-*Prkdc^scid^ Il2rg^tm1Wjl^*/SzJ) (N=6 mice/group). When tumor volumes reach ~100mm^3^ average per group, mice were randomized into treatment groups: A2780 control, A2780 plus carboplatin (50 mg/kg), A2780-HN1L control, A2780-HN1L plus carboplatin (50 mg/kg), A2780-shHN1L control, A2780-shHN1L plus carboplatin (50 mg/kg). Tumor sizes and BWs were measured biweekly. Tumor length (l) and width (w) were measured using digital calipers. Tumor volume (v) was calculated using the following equation: v=1/2*l *w^2^. Mice were sacrificed if tumors reached a diameter of 2000 mm^3^ or at the end of study. Tumor growth curves were analyzed using general linear models. The study was performed in accordance with the guidelines and standards of the Institutional Animal Care and Use Committee (IACUC) and under the approved animal protocol #11065 by Indiana University.

**Data analysis**

Prism 6 software (GraphPad, La Jolla, CA) was used to perform the statistical analysis. The associations between relative luciferase activity and relative gene expression in different groups were analyzed by Student’s t test. The data represent the means ± SD from three independent experiments. A p-value < 0.05 is considered statistically significant.

**
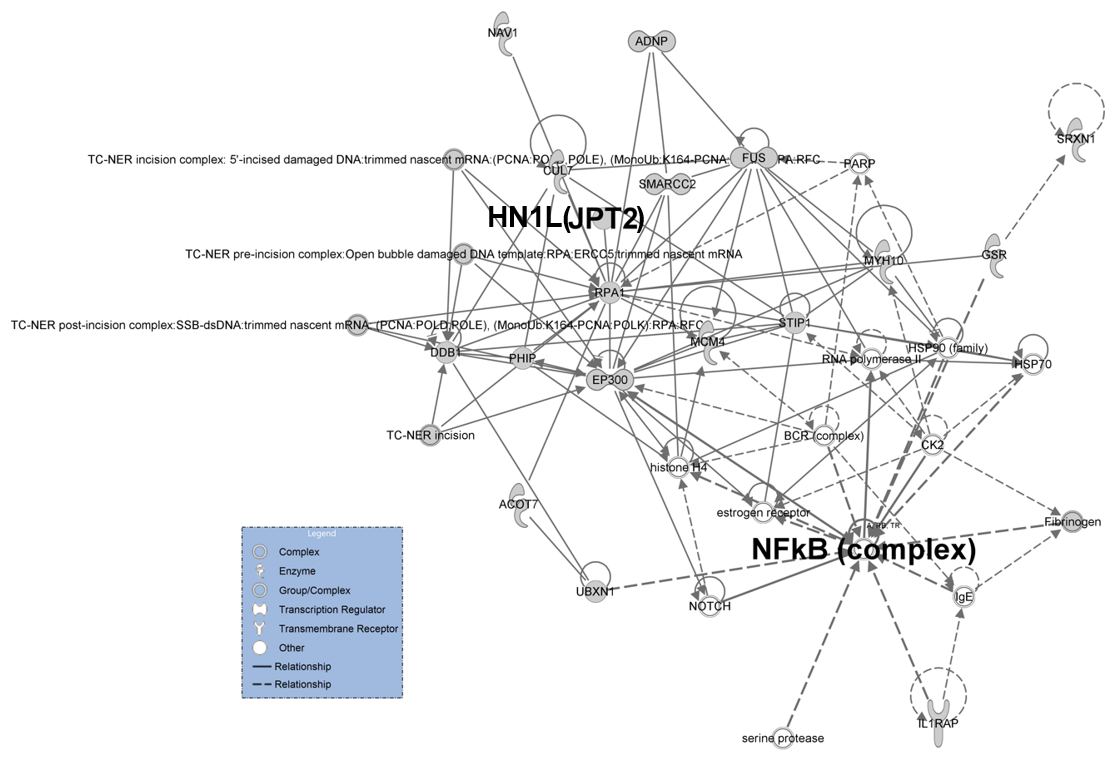
**

**Supplementary Fig. S1. IPA analysis, a representative HN1L(JPT2) related complex signaling network, with NF-κB as one of the crucial signaling node.**

**Supplementary Fig. S1.** IPA analysis, a representative HN1L(JPT2) related complex signaling network, with NF-κB as one of the crucial signaling node.
